# Hospital Meetings

**Published:** 1896-04-11

**Authors:** 


### HOSPITAL MEETINGS.

### LONDON HOMŒOPATHIC HOSPITAL.

The well-attended annual meeting of the London Homœopathic Hospital took place on March 27th. The Viscount Emlyn presided. The report, read by the Superintendent-Secretary (Mr. Cross), stated that the opening of the new buildings by H.R.H. the Duchess of Teck in June of last year had been an important event, and her kindly interest must always act as a stimulus to the work of the institution. The new wards were occupied in August, and it was evident that an enormous amount of good might be done if sufficient funds were forthcoming. They trusted to keep to the good old custom of making both ends meet at the Homœopathic Hospital. There was a little pressure on the nursing staff at present, but when the probationers now in training had completed their three years' course it was expected that there would be a sufficient number of private nurses. Miss Durning Smith referred to the heavy duties of the committee and of the hundred-and-one things necessary in new premises after the workmen are supposed to have finished. "I remember," she said, "that last year the noble treasurer (Lord Emlyn) remarked that he meant to be a working man, and he has been one." Captain Cundy said that he thought it a very great honour and privilege to have been able to labour for the hospital. It was a very home-like place, and the patients warmly appreciated their surroundings. Dr. Blackley testified that the medical staff were more than satisfied with the construction of the new building. When the business of the meeting was disposed of, the visitors visited the wards, where tea was provided for them.

